# Prehospital endotracheal intubation and return of spontaneous circulation in out-of-hospital cardiac arrest patients: a retrospective cohort study based on prehospital emergency registry data

**DOI:** 10.1186/s12873-026-01718-1

**Published:** 2026-08-11

**Authors:** Ye Yuan, Sangai Pan, Runkun Shen, Lin Wang, Songtao Shou

**Affiliations:** 1 First Branch, Tianjin Emergency Medical Center, Tianjin, 300011 China; 2 Fifth Branch, Tianjin Emergency Medical Center, Tianjin, 300011 China; 3 Department of Emergency Management, Tianjin Emergency Medical Center, Tianjin, 300011 China; 4 Department of Emergency, Tianjin Medical University General Hospital, No. 154 Anshan Road, Heping District, Tianjin, 300052 China

**Keywords:** Out-of-hospital cardiac arrest, Endotracheal intubation, Basic airway management, Return of spontaneous circulation, Prehospital emergency care, Cardiopulmonary resuscitation

## Abstract

**Background:**

The optimal airway strategy during out-of-hospital cardiac arrest (OHCA) remains uncertain. This study investigated the association between prehospital endotracheal intubation (ETI) and return of spontaneous circulation (ROSC) using a regional emergency medical services registry.

**Methods:**

This retrospective cohort study analyzed 6,162 adult non-traumatic OHCA patients treated by emergency medical services between October 2023 and November 2024. Patients were categorized into an ETI group (*n* = 1,448) and a basic airway (BA) group (*n* = 4,714). The primary outcome was prehospital ROSC. Long-term survival and neurological outcomes were not available in the registry. Multivariable logistic regression and propensity score-matched sensitivity analysis were used to evaluate the association between ETI and ROSC while accounting for measured confounders. Airway strategy was selected by on-scene physicians during routine EMS care, and rhythm-stratified unadjusted analyses were performed to describe whether the ETI-ROSC association differed by initial rhythm category.

**Results:**

ROSC occurred more frequently in the ETI group than in the BA group (21.1% vs. 3.8%, *P* < 0.001). In multivariable analysis, ETI was strongly associated with ROSC (OR = 7.61, 95% CI: 6.07–9.53, *P* < 0.001). Shockable initial rhythm (OR = 5.42, 95% CI: 3.80–7.74, *P* < 0.001) and defibrillation (OR = 1.82, 95% CI: 1.37–2.42, *P* < 0.001) were also positively associated with ROSC, whereas longer response time and epinephrine administration were negatively associated with ROSC. In 1:1 propensity score-matched analysis, the association between ETI and ROSC remained significant (OR = 6.33, 95% CI: 4.72–8.47, *P* < 0.001). Rhythm-stratified analyses showed that the unadjusted association was strongest in non-shockable and other/unknown rhythm strata and was not statistically significant in the shockable rhythm stratum.

**Conclusions:**

In this retrospective prehospital registry cohort, ETI during OHCA resuscitation was strongly associated with higher ROSC rates. Because airway timing relative to collapse and ROSC, survival to discharge, and neurological outcomes were not captured, these findings should be interpreted as an association with immediate resuscitation success rather than evidence of a causal survival benefit. The observed association may also reflect physician selection of airway strategy and other unmeasured resuscitation factors, and should therefore be considered hypothesis-generating.

**Clinical trial number:**

Not applicable.

## Introduction

Out-of-hospital cardiac arrest (OHCA) represents one of the most devastating medical emergencies worldwide, constituting a significant public health burden with substantial mortality and morbidity implications [1]. Epidemiological data indicate that approximately 350,000 OHCA events occur annually in the United States alone, with global estimates suggesting nearly 3.8 million cases per year [2]. Despite considerable advances in emergency medical services (EMS) systems and resuscitation science over the past several decades, survival rates following OHCA remain disappointingly low, with only 8% to 12% of patients surviving to hospital discharge [3]. The substantial gap between the incidence of cardiac arrest and favorable outcomes underscores the urgent need for continued research into interventions that may improve resuscitation success and long-term survival.

The pathophysiology of cardiac arrest involves the cessation of effective cardiac mechanical activity, leading to immediate cessation of systemic perfusion and rapid onset of cerebral hypoxia [4]. Within minutes of circulatory collapse, irreversible neurological injury begins to occur, making the temporal window for effective intervention extremely narrow [5]. The concept of the “chain of survival” has been established as a fundamental framework for understanding the critical interventions necessary to optimize outcomes, including early recognition and activation of emergency response systems, immediate high-quality cardiopulmonary resuscitation (CPR), rapid defibrillation for shockable rhythms, effective advanced life support, and integrated post-cardiac arrest care [6]. Among these elements, airway management has long been considered a cornerstone of advanced cardiac life support, although the optimal approach to airway intervention during cardiac arrest has remained a subject of considerable debate within the resuscitation community.

Airway management during cardiac arrest serves multiple physiological objectives, including maintenance of adequate oxygenation, prevention of hypercapnia, protection against aspiration of gastric contents, and facilitation of effective ventilation without compromising chest compression quality [7]. In prehospital resuscitation, airway strategies are usually discussed within the broader framework of advanced airway management, which in this study mainly refers to the establishment of a definitive airway such as endotracheal intubation (ETI) or the use of supraglottic airway devices [8, 9]. Traditional approaches have emphasized ETI as the classic method for securing the airway, based on its theoretical advantages in airway protection and ventilation reliability [8]. However, ETI in the prehospital setting may be technically challenging and may interrupt chest compressions if repeated attempts are required [9]. By contrast, supraglottic airway devices are generally easier to insert and may provide a practical alternative in selected resuscitation scenarios. Thus, in the present study, supraglottic airway devices are mentioned as one possible form of advanced airway management, rather than as a separate research topic.

The theoretical framework supporting advanced airway management during cardiac arrest is based on the principle that adequate oxygenation and ventilation are essential components of successful resuscitation [10]. During CPR, cardiac output is substantially reduced compared to normal physiological conditions, typically achieving only 25–30% of baseline values even with optimal chest compression technique [11]. Under these circumstances, effective ventilation becomes critical for maintaining oxygen delivery to vital organs, particularly the brain and heart, which are highly sensitive to hypoxic injury [12]. Advanced airway devices theoretically provide more reliable ventilation compared to bag-valve-mask (BVM) ventilation alone, which requires continuous manual maintenance of an adequate seal and may be associated with gastric insufflation and subsequent aspiration risk [13]. Furthermore, advanced airways enable continuous chest compressions without pauses for ventilation, potentially improving coronary perfusion pressure and the probability of successful defibrillation.

Despite the compelling theoretical rationale for advanced airway management, empirical evidence regarding its impact on patient-centered outcomes has been inconsistent [9, 13–17]. In the AIRWAYS-2 randomized trial, a supraglottic airway strategy did not significantly improve favorable neurological outcome compared with tracheal intubation [9, 17]. In the PART trial, initial laryngeal tube insertion improved 72-hour survival compared with endotracheal intubation, but the reported effect size was substantially smaller than the association observed in many retrospective analyses [16]. Large observational studies have also produced heterogeneous results, partly because airway intervention, resuscitation duration, provider skill, and patient selection are difficult to separate in registry data [15, 18–20].

A significant methodological challenge in studying the relationship between airway management and cardiac arrest outcomes is resuscitation time bias. Patients must survive long enough during the resuscitation attempt to receive an airway intervention; therefore, patients who receive ETI are not directly comparable with those who rapidly achieve ROSC or are declared dead before ETI can be attempted [18, 21]. This bias can inflate the apparent benefit of ETI if the timing of collapse, airway placement, drug administration, defibrillation, and ROSC cannot be modeled explicitly. Studies based on retrospective registries therefore require cautious causal language, adjustment for measured confounders, and sensitivity analyses that test whether the observed association is robust to alternative analytic assumptions.

The current study examined the association between prehospital ETI and ROSC in a large regional cohort of OHCA patients. The analysis focused on ETI because the registry reliably recorded tracheal intubation, bag-valve-mask ventilation, and oropharyngeal airway use, whereas supraglottic airway placement was not captured as a separate analyzable device category. We also evaluated measured confounding through multivariable regression and propensity score matching, while recognizing that residual confounding and resuscitation time bias cannot be fully eliminated without precise time-to-intervention data.

## Methods

### Study design and setting

This retrospective cohort study utilized data from a regional prehospital emergency care registry encompassing emergency medical services missions conducted between October 2023 and November 2024. This interval was selected because the electronic registry fields for airway intervention, cardiac rhythm, emergency time stamps, medication use, defibrillation, and ROSC were consistently available across the regional system during this period. The registry prospectively records standardized prehospital care variables entered by EMS physicians and audited within the Tianjin Emergency Medical Center system. The regional EMS system is physician-staffed; however, the registry did not assign airway strategy by a research protocol. During routine resuscitation, the on-scene emergency physician selected ETI or continued bag-valve-mask ventilation according to the patient’s clinical status, airway assessment, expected resuscitation needs, and provider judgment within local EMS practice.

### Study population

The study population consisted of adult patients who received cardiopulmonary resuscitation (CPR) or had a primary diagnosis of non-traumatic cardiac arrest recorded in the emergency registry database. Patients were eligible for inclusion if they were 18 years or older, experienced OHCA, received EMS resuscitation, and had complete documentation of airway strategy, key interventions, emergency response time, and ROSC status.

Inclusion Criteria: Age > = 18 years; Non-traumatic out-of-hospital cardiac arrest; Resuscitation attempted by EMS personnel; Complete documentation of airway management strategy; Available outcome data regarding ROSC status; Complete core intervention records for epinephrine administration and defibrillation.

Exclusion Criteria: Traumatic cardiac arrest; Pediatric patients (< 18 years); Missing data on the primary outcome (ROSC); Missing data on airway management approach; Missing core intervention documentation; Do-not-resuscitate orders documented prior to EMS arrival.

The raw registry contained 6,171 eligible adult non-traumatic OHCA mission records. The predefined complete-case analytic cohort comprised 6,162 patients with complete outcome, airway, and key intervention documentation, including 1,448 patients in the ETI group and 4,714 patients in the BA group.

### Airway exposure definition

Patients were categorized into two groups based on the documented airway strategy used during prehospital resuscitation:

Endotracheal Intubation Group (ETI Group, *n* = 1,448): Patients who received documented tracheal intubation during prehospital resuscitation.

Basic Airway Group (BA Group, *n* = 4,714): Patients who received bag-valve-mask ventilation with or without an oropharyngeal airway adjunct and had no documented endotracheal intubation. Because supraglottic airway placement was not separately and consistently coded in the registry, device-specific subgroup analysis for supraglottic airways was not performed. The recorded exposure therefore represents the airway approach actually used during care rather than randomized or protocol-mandated assignment, and the registry did not capture the detailed clinical rationale for selecting ETI versus continued basic airway management.

### Data collection and variables

Data were extracted from the electronic emergency registry database and included demographic variables (age and sex), medical history (hypertension, heart disease, diabetes mellitus, and cerebrovascular disease), emergency response variables (EMS response time), initial cardiac rhythm (shockable, non-shockable, or other/unknown), airway intervention (ETI, bag-valve-mask ventilation, and oropharyngeal airway use), and resuscitation interventions (epinephrine administration and defibrillation). The primary outcome was prehospital ROSC. Survival to hospital discharge and neurological outcome were not available in the registry dataset.

### Instruments and measurements

All cardiac rhythm assessments were performed using standard cardiac monitors/defibrillators (Mindray Integrated Defibrillator Monitor) with rhythm interpretation performed directly by on-scene emergency physicians. Response time was defined as the interval from EMS dispatch to arrival at the scene. Because response-time values were right-skewed and contained operational outliers, response time was summarized using the median and interquartile range for descriptive analyses and entered as a continuous covariate in regression after data cleaning. ROSC was defined as the restoration of palpable pulse and organized circulation as documented by the treating EMS team before hospital handover.

### Ethical considerations

This study was approved by the Ethics Committee of Tianjin Emergency Medical Center (No. TJEMC-2023-003) and was conducted in accordance with the principles of the Declaration of Helsinki. The Ethics Committee of Tianjin Emergency Medical Center waived the requirement for informed consent to participate because this retrospective study used de-identified registry data.

### Statistical analysis

Statistical analyses were performed using IBM SPSS Statistics version 26.0 (IBM Corporation, Armonk, NY, USA) and R version 4.3. Continuous variables were assessed for distributional characteristics and are presented as mean ± standard deviation or median [interquartile range], as appropriate. Categorical variables are presented as frequencies and percentages. Between-group comparisons were performed using Student’s t test or the Mann-Whitney U test for continuous variables and the chi-square test for categorical variables. Multivariable logistic regression was used to estimate the association between ETI and ROSC after adjustment for age, sex, response time, epinephrine administration, defibrillation, and shockable initial rhythm. Model performance was evaluated using the Hosmer-Lemeshow goodness-of-fit test and the area under the receiver operating characteristic curve (AUC). To assess robustness against measured baseline imbalance, 1:1 nearest-neighbor propensity score matching without replacement was performed using age, sex, comorbidities, response time, and initial rhythm category. Covariate balance was evaluated using standardized mean differences, and *P* < 0.05 was considered statistically significant. Because initial rhythm could modify the unadjusted relationship between airway strategy and ROSC, rhythm-stratified 2 × 2 analyses were conducted for non-shockable, shockable, and other/unknown rhythm strata. For each stratum, ROSC counts, unadjusted odds ratios, 95% confidence intervals, and P-values were calculated using the basic airway group as the reference.

## Results

### Baseline characteristics

The baseline demographic and clinical characteristics of the study population stratified by airway management group are presented in Table 1. A total of 6,162 patients met the inclusion criteria, with 1,448 (23.5%) in the ETI group and 4,714 (76.5%) in the BA group.

**Table 1:** Comparison of baseline characteristics between study groups

Variable	Basic Airway Group (*n* = 4,714)	ETI Group (*n* = 1,448)	*P*-value
Age (years, Mean ± SD)	70.3 ± 14.4	69.2 ± 13.2	0.004
Male sex, n (%)	3,109 (66.0%)	1,011 (69.8%)	0.007
Hypertension, n (%)	976 (20.7%)	314 (21.7%)	0.444
Heart disease, n (%)	1,353 (28.7%)	481 (33.2%)	0.001
Diabetes mellitus, n (%)	663 (14.1%)	251 (17.3%)	0.003
Cerebrovascular disease, n (%)	806 (17.1%)	248 (17.1%)	1.000

As shown in Table 1, patients in the ETI group were slightly younger than those in the BA group (69.2 ± 13.2 years vs. 70.3 ± 14.4 years, *P* = 0.004) and had a higher proportion of male patients (69.8% vs. 66.0%, *P* = 0.007). Heart disease and diabetes mellitus were more frequent in the ETI group, whereas hypertension and cerebrovascular disease showed no clinically meaningful between-group difference.

### Emergency response and cardiac arrest characteristics

**Fig. 1:**
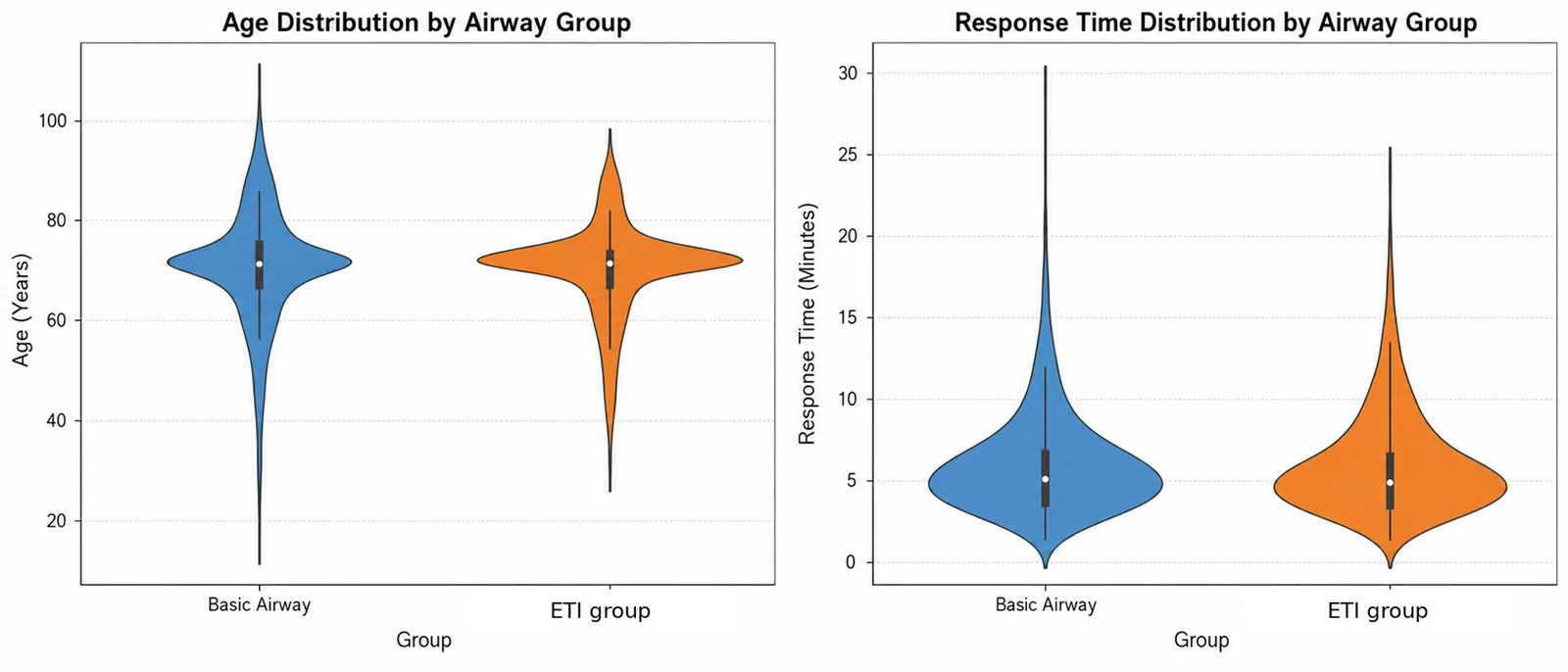
Violin plots demonstrating age distribution and response-time distribution by airway management group, illustrating the registry distribution of these continuous variables

The prehospital emergency response characteristics and cardiac arrest parameters are summarized in Table 2. EMS response time was comparable between groups when summarized using the median and interquartile range (5.6 [4.0-7.4] min in the ETI group vs. 5.5 [4.1–7.4] min in the BA group, *P* = 0.829). The use of median [interquartile range] better reflected the skewed operational distribution of response-time data than mean ± standard deviation. Figure 1 illustrates the distributions of age and response time by airway management group.

**Table 2:** Comparison of prehospital emergency response characteristics

Variable	Basic Airway Group	ETI Group	*P*-value
Response time (min, Median [IQR])	5.5 [4.1–7.4]	5.6 [4.0-7.4]	0.829
Initial cardiac rhythm, n (%)			0.003
Non-shockable (PEA/Asystole)	3,207 (68.0%)	980 (67.7%)	
Shockable (VF/VT)	209 (4.4%)	95 (6.6%)	
Other/Unknown	1,298 (27.5%)	373 (25.8%)	

The distribution of initial cardiac rhythm differed significantly between groups (*P* = 0.003). Shockable rhythms were documented in 6.6% of patients in the ETI group and 4.4% of patients in the BA group. A substantial proportion of records were categorized as other/unknown rhythm, which reflects the limitations of real-world prehospital registry documentation and was considered when interpreting rhythm-stratified results.

### Resuscitation interventions

Analysis of resuscitation interventions revealed substantial differences between groups in the intensity and type of advanced life support measures received, as detailed in Table 3.

**Table 3:** Comparison of resuscitation interventions between groups

Variable	Basic Airway Group, *n* (%)	ETI Group, *n* (%)	*P*-value
Epinephrine administration	3,310 (70.2%)	1,396 (96.4%)	< 0.001
Defibrillation performed	482 (10.2%)	268 (18.5%)	< 0.001
Bag-valve-mask ventilation	3,975 (84.3%)	6 (0.4%)	< 0.001
Oropharyngeal airway	738 (15.7%)	2 (0.1%)	< 0.001
Endotracheal intubation	0 (0.0%)	1,448 (100.0%)	< 0.001

Patients in the ETI group received higher rates of epinephrine administration (96.4% vs. 70.2%, *P* < 0.001) and defibrillation (18.5% vs. 10.2%, *P* < 0.001). In contrast, bag-valve-mask ventilation and oropharyngeal airway use were concentrated in the BA group by definition.

### Primary outcome: return of spontaneous circulation

**Fig. 2:**
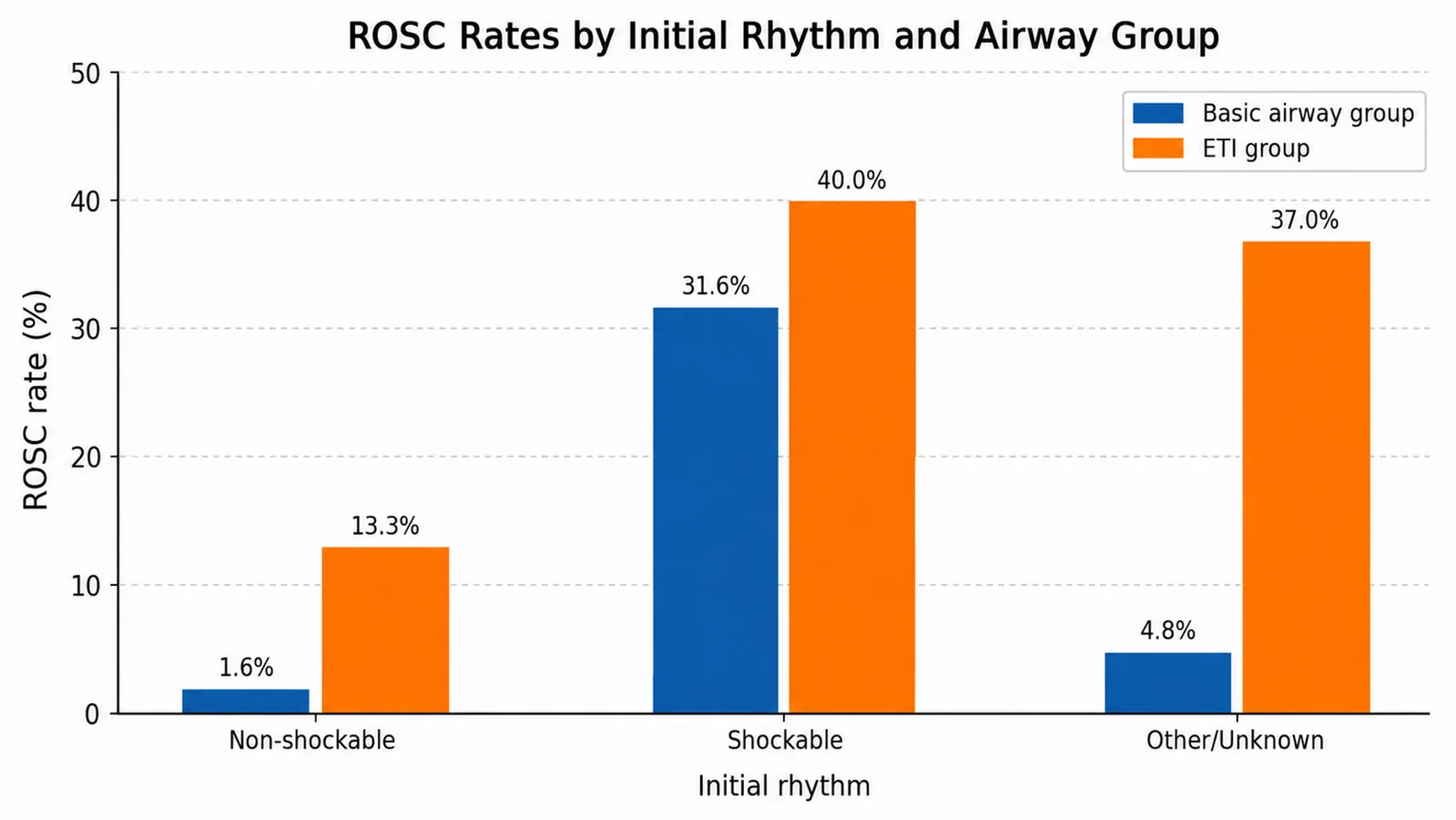
Bar chart displaying ROSC rates stratified by initial cardiac rhythm category and airway management strategy, using the categories non-shockable, shockable, and other/unknown

The primary outcome of ROSC was achieved in a significantly higher proportion of patients in the ETI group than in the BA group (306/1,448 [21.1%] vs. 178/4,714 [3.8%], *P* < 0.001). ROSC rates stratified by initial cardiac rhythm and airway management strategy are presented in Fig. 2.

In rhythm-stratified analyses, the ETI-ROSC association was most pronounced among patients with non-shockable rhythms, with ROSC in 130/980 ETI patients (13.3%) compared with 50/3,207 BA patients (1.6%), corresponding to an unadjusted OR of 9.66 (95% CI: 6.91–13.50, *P* < 0.001). In the shockable rhythm stratum, ROSC occurred in 38/95 ETI patients (40.0%) and 66/209 BA patients (31.6%), yielding a smaller and statistically non-significant association (OR = 1.44, 95% CI: 0.87–2.39, *P* = 0.151). In the other/unknown rhythm stratum, ROSC occurred in 138/373 ETI patients (37.0%) and 62/1,298 BA patients (4.8%), with an unadjusted OR of 11.71 (95% CI: 8.41–16.29, *P* < 0.001). These rhythm-stratified findings are reported in Table 4.

**Table 4:** Rhythm-stratified unadjusted association between ETI and ROSC

Initial rhythm stratum	ETI ROSC/total (%)	BA ROSC/total (%)	Unadjusted OR (95% CI; *P*-value)
Non-shockable (PEA/Asystole)	130/980 (13.3%)	50/3,207 (1.6%)	9.66 (6.91–13.50; *P* < 0.001)
Shockable (VF/VT)	38/95 (40.0%)	66/209 (31.6%)	1.44 (0.87–2.39; *P* = 0.151)
Other/Unknown	138/373 (37.0%)	62/1,298 (4.8%)	11.71 (8.41–16.29; *P* < 0.001)

**Fig. 3:**
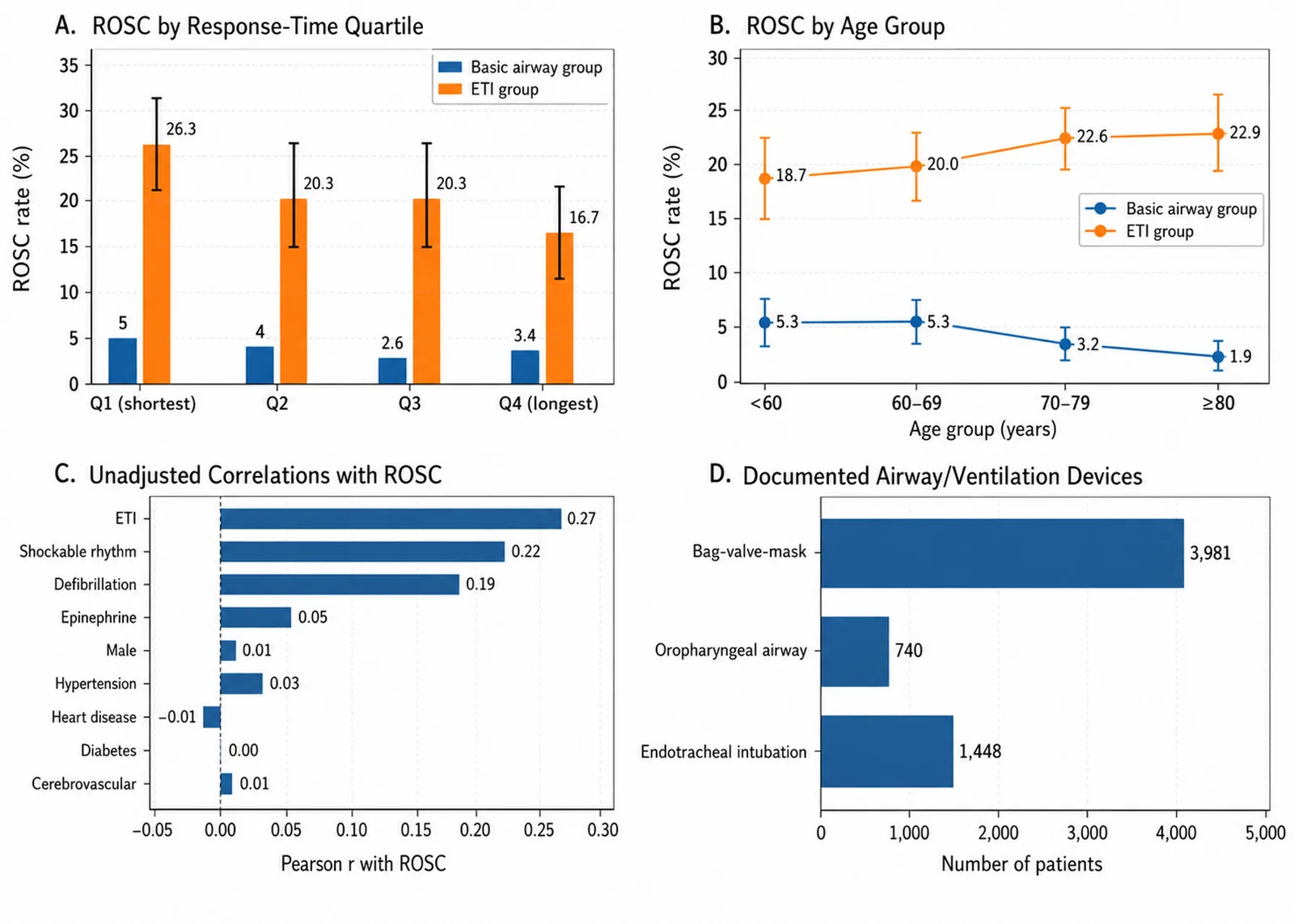
Multi-panel figure showing (top left) ROSC rate by response-time quartile stratified by airway group, (top right) ROSC trends by age group, (bottom left) unadjusted associations between selected clinical factors and ROSC, and (bottom right) documented airway intervention patterns

Exploratory descriptive analyses showed that ROSC varied by response-time quartile, age group, rhythm category, and airway intervention pattern (Fig. 3). These unadjusted patterns were used to describe the registry cohort and generate clinical context; the primary inference was based on multivariable regression and propensity score-matched sensitivity analysis rather than on unadjusted correlations.

### Multivariate logistic regression analysis

**Fig. 4:**
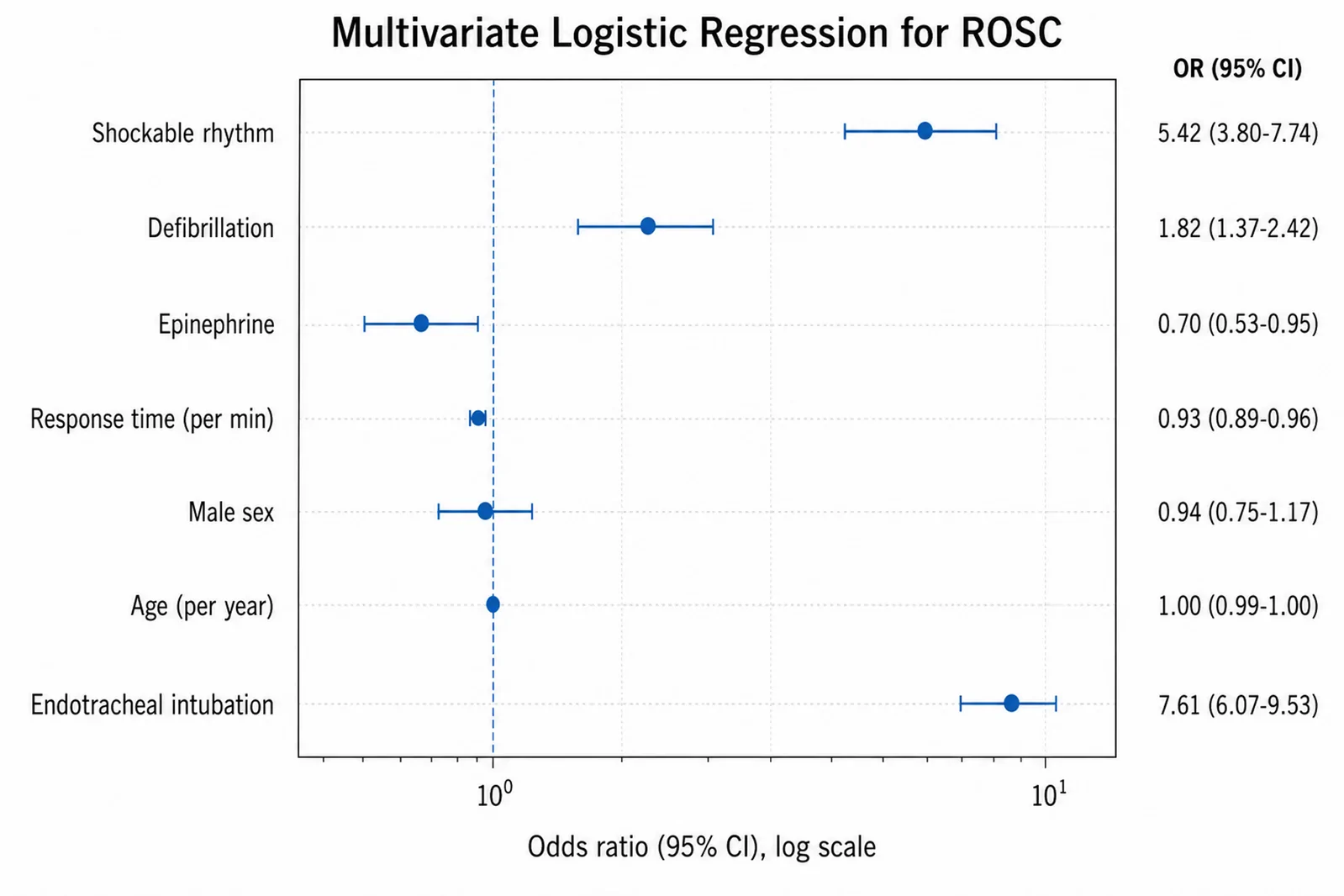
Forest plot displaying the multivariable logistic regression results with odds ratios and 95% confidence intervals for variables included in the model predicting ROSC; estimates correspond to Table 5

To identify factors associated with ROSC while controlling for measured confounding variables, multivariable logistic regression analysis was performed. The results are presented in Table 5; Fig. 4.

**Table 5:** Multivariable logistic regression analysis for predictors of ROSC

Variable	Coefficient (β)	SE	*P*-value	OR (95% CI)
Endotracheal intubation (Yes vs. No)	2.029	0.115	< 0.001	7.61 (6.07–9.53)
Age (per year increase)	-0.004	0.004	0.310	1.00 (0.99-1.00)
Male sex	-0.065	0.114	0.568	0.94 (0.75–1.17)
Response time (per minute)	-0.075	0.019	< 0.001	0.93 (0.89–0.96)
Epinephrine administration	-0.350	0.150	0.020	0.70 (0.53–0.95)
Defibrillation performed	0.598	0.146	< 0.001	1.82 (1.37–2.42)
Shockable initial rhythm	1.690	0.182	< 0.001	5.42 (3.80–7.74)

As demonstrated in Table 5; Fig. 4, ETI remained strongly associated with ROSC after adjustment for all covariates (OR = 7.61, 95% CI: 6.07–9.53, *P* < 0.001). Shockable initial rhythm was also positively associated with ROSC (OR = 5.42, 95% CI: 3.80–7.74, *P* < 0.001), as was defibrillation (OR = 1.82, 95% CI: 1.37–2.42, *P* < 0.001). Conversely, each additional minute of response time was associated with a lower probability of ROSC (OR = 0.93, 95% CI: 0.89–0.96, *P* < 0.001). Epinephrine administration showed a negative association with ROSC in the adjusted model (OR = 0.70, 95% CI: 0.53–0.95, *P* = 0.020), which is most consistent with confounding by indication because epinephrine is generally administered in more prolonged or refractory resuscitation attempts.

The Hosmer-Lemeshow test indicated acceptable model calibration (chi-square = 8.24, *P* = 0.412), and the model demonstrated good discrimination with an AUC of 0.802. In the 1:1 propensity score-matched sensitivity analysis, 1,438 matched pairs were obtained and all post-matching standardized mean differences were below 0.10. ROSC occurred in 302/1,438 (21.0%) matched ETI patients and 58/1,438 (4.0%) matched BA patients. The association between ETI and ROSC remained significant after matching (OR = 6.33, 95% CI: 4.72–8.47, *P* < 0.001), supporting robustness to measured baseline imbalance while not eliminating the possibility of resuscitation time bias or unmeasured confounding (Table 6).

**Table 6:** Propensity score-matched sensitivity analysis for the association between ETI and ROSC

Matched analysis item	Result
Matched pairs	1,438
ROSC in matched BA patients	58/1,438 (4.0%)
ROSC in matched ETI patients	302/1,438 (21.0%)
ETI association with ROSC	OR = 6.33 (95% CI: 4.72–8.47), *P* < 0.001
Post-matching covariate balance	All standardized mean differences < 0.10

## Discussion

The present study found a strong association between prehospital ETI and ROSC among adult OHCA patients treated within a regional EMS system. In the adjusted model, ETI was associated with substantially higher odds of ROSC (OR = 7.61, 95% CI: 6.07–9.53), and this association persisted in propensity score-matched analysis. However, this finding should be interpreted cautiously. The outcome was limited to prehospital ROSC, and the registry did not capture survival to discharge or neurological outcome. Therefore, the results support an association with immediate resuscitation success rather than a causal survival benefit.

The magnitude of the association observed in this study is notably higher than the effect sizes reported in major randomized airway trials. The AIRWAYS-2 trial compared supraglottic airway management with tracheal intubation and found no significant difference in favorable neurological outcome at 30 days [9, 17]. The PART trial compared initial laryngeal tube insertion with endotracheal intubation and reported improved 72-hour survival in the laryngeal tube group, but the trial did not demonstrate an effect size comparable to the adjusted OR observed in this registry analysis [16]. These differences are clinically important because randomized trials typically evaluate patient-centered outcomes and reduce treatment-selection bias, whereas retrospective registries may overestimate intervention effects when timing and treatment intensity are incompletely measured.

First, resuscitation time bias is the most important threat to interpretation. Patients who receive ETI must remain under active resuscitation long enough for an intubation attempt to occur. Patients with very early ROSC may not require ETI, whereas patients with rapid termination of resuscitation may not have the opportunity to receive ETI. This creates a time-dependent exposure problem in which ETI can appear strongly protective even when part of the association reflects survival time before exposure [18, 21]. Exact time stamps for airway placement, collapse-to-ROSC interval, and termination of resuscitation were not available in the registry, so landmark analysis or time-dependent covariate modeling could not be performed. Propensity score matching reduced measured baseline imbalance, but it cannot correct for unmeasured resuscitation duration or intervention timing.

Second, ETI was closely linked with more intensive resuscitation. Patients in the ETI group received substantially more epinephrine and defibrillation, suggesting longer or more aggressive resuscitation efforts. In the adjusted model, epinephrine administration was negatively associated with ROSC, which should not be interpreted as a direct harmful effect in isolation. Rather, epinephrine use probably identifies patients with refractory arrest, longer resuscitation, or poorer early response to CPR [22, 23]. Defibrillation remained positively associated with ROSC, consistent with its central role in shockable rhythms, but the co-occurrence of ETI, epinephrine, and defibrillation illustrates why the independent contribution of airway management is difficult to isolate in observational data.

Third, the association between ETI and ROSC may partly reflect local EMS practice and patient selection. The study was conducted in a single regional EMS system in which airway placement was performed by trained emergency physicians and recorded in a standardized registry. Systems with different staffing models, airway training, response intervals, transport practices, or supraglottic airway protocols may observe different associations. The restriction to records from October 2023 to November 2024 also reflects the period in which core registry variables were consistently available, but it limits temporal generalizability.

A related but distinct concern is physician selection bias in the airway decision itself. In this physician-staffed EMS system, the decision to perform ETI may have incorporated bedside prognostic impressions, perceived reversibility, airway anatomy, aspiration risk, rhythm evolution, response to early CPR, family preference, and anticipated transport needs. These factors are not fully represented by age, sex, comorbidity history, response time, initial rhythm, epinephrine use, or defibrillation, and therefore could produce residual differences between ETI and BA patients even after multivariable adjustment and propensity score matching. This mechanism differs from resuscitation time bias because it concerns why ETI was selected, not only whether the patient survived long enough for ETI to be attempted.

From a mechanistic perspective, ETI may offer several advantages during selected resuscitation scenarios. Tracheal intubation can secure the airway, reduce aspiration risk, and permit ventilation without repeated mask repositioning. When performed efficiently, it may also support continuous chest compressions without prolonged ventilation pauses, potentially preserving coronary perfusion pressure and improving the chance of successful defibrillation [24–26]. These theoretical advantages depend heavily on operator skill and on avoiding interruptions in chest compressions, because intubation attempts that prolong pauses or delay defibrillation could offset any airway benefit [27].

Shockable initial rhythm remained an important predictor of ROSC, which aligns with prior evidence that ventricular fibrillation and pulseless ventricular tachycardia are more responsive to early defibrillation than asystole or pulseless electrical activity [28, 29]. The other/unknown rhythm category was common in this real-world registry, reflecting incomplete or heterogeneous documentation of initial rhythm during time-critical care. This category should not be interpreted as a biologically uniform rhythm group. Instead, it indicates that registry-based rhythm analyses require cautious interpretation and that future work should improve standardized rhythm capture. The rhythm-stratified analysis further supports cautious interpretation: the ETI association was large in non-shockable and other/unknown rhythms but much smaller and statistically non-significant in shockable rhythms, where early defibrillation provides a competing pathway to ROSC that is less dependent on airway intervention.

The negative association between response time and ROSC reinforces the time-critical nature of OHCA management [30, 31]. Response time was similar between airway groups when summarized as median [interquartile range], reducing concern that the ETI group simply received faster EMS arrival. Nevertheless, the registry did not reliably capture witnessed arrest, bystander CPR, no-flow time, low-flow time, or exact collapse-to-ROSC duration, all of which are major determinants of outcome and may confound the observed association between ETI and ROSC.

The clinical implication of these findings is not that ETI should be universally prioritized over other airway strategies in all OHCA patients. Rather, ETI appears to be associated with higher prehospital ROSC in this EMS system when performed as part of a broader advanced life support package. Decisions regarding airway strategy should continue to balance oxygenation, aspiration risk, provider proficiency, chest-compression continuity, and timely defibrillation. The findings support further prospective evaluation of which patient subgroups may benefit from ETI and when airway placement should occur during the resuscitation sequence. The results should therefore not be interpreted as evidence that ETI itself caused ROSC, but as evidence that documented ETI was associated with ROSC in a physician-led EMS context where airway choice was clinically selected.

Several limitations of this study warrant consideration. First, the retrospective observational design precludes definitive causal inference, and unmeasured confounding may influence the observed associations. Second, resuscitation time bias could not be fully addressed because exact airway placement time, collapse-to-ROSC interval, and resuscitation termination time were unavailable. Third, the primary outcome was limited to prehospital ROSC; survival to hospital discharge and neurological outcomes were not captured. Fourth, witnessed status, bystander CPR, no-flow time, low-flow time, and detailed airway-attempt characteristics were not reliably available. Fifth, supraglottic airway placement was not separately coded, so the analysis specifically evaluates documented ETI rather than all advanced airway devices. Sixth, the single-region setting and the limited registry period may restrict generalizability to other EMS systems. Seventh, airway strategy was chosen by on-scene physicians during real-world resuscitation rather than assigned by protocol, so physician selection bias may remain even after adjustment for measured covariates.

In conclusion, this large retrospective cohort study showed that documented prehospital ETI was strongly associated with higher ROSC rates among adult patients with OHCA. The association remained significant after adjustment and propensity score matching, but the observational design, lack of precise airway timing, and absence of survival and neurological outcomes require cautious interpretation. Future prospective studies should incorporate exact intervention timing, witnessed status, bystander CPR, survival to discharge, and neurological outcomes to determine whether ETI improves patient-centered outcomes and to identify the optimal timing and patient subgroups for airway intervention. The rhythm-stratified findings and physician-discretion exposure mechanism emphasize that the results should be interpreted as associations within this EMS system rather than as definitive evidence favoring routine ETI for all OHCA patients.

## Data Availability

Data supporting the findings of this study are available from the corresponding author upon reasonable request.
